# Advanced Glycation End Products and Mobility Decline: A Novel Perspective on Aging

**DOI:** 10.3390/healthcare13060613

**Published:** 2025-03-12

**Authors:** Hyeong Jun Park, Moon Jin Lee, Jiyoun Kim

**Affiliations:** 1 Department of Physical Therapy, College of Health Science, Gachon University, Incheon 21936, Republic of Korea; phj2024@gachon.ac.kr; 2 Department of Exercise Rehabilitation, Institute of Human Convergence Health Science, Gachon University, Incheon 13120, Republic of Korea; mjlee01@korea.ac.kr; 3 Department of Physical Education, College of Education, Korea University, Seoul 02841, Republic of Korea

**Keywords:** mobility decline, advanced glycation end products, skin auto-fluorescence, older adults, sit-to-stand, gait speed, single-leg stance, Timed Up and Go

## Abstract

**Background/Objectives**: Advanced Glycation End Products (AGEs) are high-molecular-weight compounds formed through non-enzymatic reactions between sugars and proteins, lipids, or nucleic acids. This study aimed to comprehensively analyze the association between the accumulation of AGEs and lower-limb muscle strength, gait speed, and balance abilities related to mobility in elderly individuals. **Methods**: This cross-sectional correlational study included 552 community-dwelling older adults. AGE accumulation was assessed using skin autofluorescence (SAF) measured using an AGE reader. Mobility decline factors were evaluated using the sit-to-stand (STS), gait speed (4 m walk tests), single-leg stance (SLS), and Timed Up and Go (TUG) tests. **Results**: A comparison of the physical function across the quartile groups revealed that the group with the highest SAF values, Q4, exhibited a general decline in STS, gait speed, SLS, and TUG performance when compared with the other groups (*p* < 0.001). Spearman’s correlation analysis revealed that the SAF-AGEs demonstrated significant negative correlations with STS (*r* = −0.211, *p* < 0.001), gait speed (*r* = −0.243, *p* < 0.001) and SLS (*r* = −0.201, *p* < 0.001). Additionally, TUG showed a significant positive correlation (*r* = 0.239, *p* < 0.001). In the logistic regression analysis, compared with the Q1 group, the Q4 group had significantly higher odds of low STS performance (odds ratio (OR) = 2.43, *p* = 0.006), slow gait speed (OR = 2.28, *p* = 0.002), low SLS performance (OR = 2.52, *p* = 0.001), and slow TUG (OR = 2.00, *p* = 0.035). The optimal cutoff value of the SAF for mobility decline was 3.15 (area under the curve 0.694; 95% confidence interval: 0.618–0.771). **Conclusions**: This study has demonstrated that higher SAF values were associated with decreased lower-limb strength, gait speed, and balance, thereby suggesting that SAF may be a useful screening tool for predicting mobility decline in older adults.

## 1. Introduction

With the rapid progression of global aging, mobility in older adults has garnered considerable attention as a crucial factor for independent living and enhancing the quality of life [1,2]. Mobility refers to an individual’s ability to move safely and independently from one location to another [3]. A decline in mobility not only leads to reduced physical function, increased risk of falls, and difficulties in performing daily activities, but also exacerbates the social and economic burden on the elderly population [4,5]. The primary causes of this decline include decreased lower-limb muscle strength and reduced balance ability [6,7], both of which are essential for generating movement and maintaining an upright posture during mobility [8]. Therefore, maintaining lower-limb muscle strength and balance is crucial for preventing mobility decline and reducing the risk of falls in older adults.

Recent studies have identified advanced glycation end products (AGEs) as significant contributors to the decline in physical functions [8,9,10]. AGEs are high-molecular-weight compounds formed through non-enzymatic reactions between sugars and proteins, lipids, or nucleic acids [11]. They accumulate in the body over prolonged periods, inducing structural alterations and functional impairments in musculoskeletal and neural tissues, thereby negatively affecting overall physical functions, including gait [12,13,14]. Consequently, inhibiting AGE accumulation can be considered crucial for maintaining mobility and preventing a decline in physical function in older adults [15].

AGEs can be non-invasively assessed using skin autofluorescence (SAF); the SAF measurement method using an AGE reader is considered simpler and less burdensome for participants than existing invasive methods [16]. Previous studies have reported that SAF values exhibit negative correlations with major physical function indicators, such as grip strength, lower-limb muscle strength decline, and gait speed reduction [17,18]. However, maintaining mobility requires not only lower-limb muscle strength, but also static and dynamic balance abilities [19]. Existing studies have not comprehensively analyzed lower limb functions related to mobility. Therefore, to clearly elucidate the association between SAF and mobility in the elderly, studies analyzing a more diverse range of lower-limb functions are warranted.

In addition, the proportion of individuals aged 65 and older in South Korea has rapidly increased, accounting for approximately 19.2% of the total population [20]. This study was conducted in two urban areas with a high distribution of older adults, where AGEs accumulation is expected to be more pronounced. On the other hand, the increase in oxidative stress with aging may further contribute to AGEs formation. Considering these demographic characteristics, investigating the relationship between AGEs and mobility decline in this specific population is essential for developing targeted interventions.

Thus, this study aimed to comprehensively analyze the association between the accumulation of AGEs and lower-limb muscle strength, gait speed, and balance abilities related to mobility in older adults. To achieve this, we employed the sit-to-stand (STS), 4 m walk, single-leg stance (SLS), and Timed Up and Go (TUG) tests to evaluate lower-limb function. Furthermore, this study explored the potential use of AGEs as predictive indicators of mobility decline in older adults.

## 2. Materials and Methods

### 2.1. Study Design

This cross-sectional correlational study was conducted among community-dwelling older adults. This study aimed to investigate the association between SAF and mobility decline in older adults based on the SAF measurements, lower-limb muscle strength, gait speed, and balance ability.

### 2.2. Study Participants

**Figure 1:**
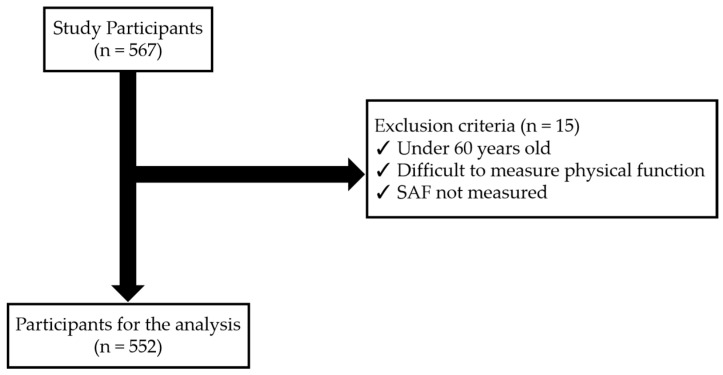
Flowchart of the subjects in the study.

This study recruited 567 elderly individuals aged between 60 and 100 years living in City I and City S, South Korea, through a voluntary sampling method from February to June 2023. The selection criteria for participants were as follows:

Participants were excluded from the study if they were under 60 years of age, had difficulties in measuring physical functions due to orthopedic diseases, or if SAF measurements were not conducted. After applying these exclusion criteria, a final sample of 552 individuals was included in the analysis (Figure 1).

All study procedures were approved by the Gachon University Institutional Bioethics Committee (Approval Number: 1044396-202301-HR-019-01), and the study was conducted in accordance with the principles of the Declaration of Helsinki.

### 2.3. SAF Measurement

In this study, SAF was measured using an AGE Reader™ (DiagnOptics Technologies BV, Groningen, The Netherlands), a device that non-invasively assesses accumulated AGEs in the skin. During the measurement process, excitation light with a wavelength of 370 nm was directed onto a 4 cm^2^ area of the skin on the dominant forearm, and the reflected autofluorescence was analyzed. The SAF analyzer calculated the skin AGE levels based on the intensity of the reflected light, and the results were recorded in arbitrary units (AU) [21].

### 2.4. Measurement of Lower Limb Strength

Lower-limb muscle strength was measured as the number of STS repetitions performed by the participants for over 30 s while sitting on a chair without armrests, with the hips and knees flexed at 90°. Participants were instructed to cross their arms over their chest to secure them and began the assessment upon receiving the instructions “ready” and “start”. At this time, a complete STS from the seated position was counted as one repetition [22]. Low STS performance was defined as the lowest 25th percentile in both sexes.

### 2.5. Measurement of Gait Speed

Gait speed was measured using the 4 m walk test. A 4 m distance was measured and marked on the floor with tape to indicate the start and end points. Participants were instructed to begin walking from a standing position at their usual walking speed and maintain a normal pace until they passed the endpoint. The time was recorded from the moment the first step was taken until the foot touched the ground at the end point [23]. A slow gait speed was defined as a walking speed of 0.8 m/s or less. This is regarded as a pathological gait speed that is closely associated with mobility difficulties and an increased risk of falls [24].

### 2.6. Static Balance Measurement

Static balance was assessed using the SLS test on a flat surface. The participants were instructed to place their hands on their hips, bend one knee so that the foot was lifted off the ground, and maintain balance on one leg for a maximum of 60 s. The duration was measured separately for the left and right legs, and the test was terminated when the lifted foot touched the ground or when the hands left the hips. The final recorded time was the longest duration between the two legs [25]. Low SLS performance was defined as an SLS duration of ≤5 s, which is recognized as an indicator associated not only with a decline in balance abilities but also with increased mortality rates [26].

### 2.7. Dynamic Balance Measurement

Dynamic balance was measured using the TUG test. The participants were instructed to rise from a chair with a seat height of 50 cm, place their backs against the chair’s backrest, walk 3 m at a normal walking speed to circle a traffic cone, and then return to sit back on the chair. Time measurement commenced with the instruction “start” and concluded when the participant was fully seated with their back touching the chair’s backrest [27]. Low TUG performance was defined as a TUG completion time of ≥13.5 s, which has been reportedly associated with a decline in balance and an increased risk of falls [28].

### 2.8. Statistical Analysis

The collected data were analyzed using SPSS 24 (IBM Corporation, Armonk, NY, USA). Variables are presented as mean ± standard deviation or frequency. Differences between groups for continuous variables were analyzed using the Kruskal–Wallis test. Categorical variables are expressed as percentages and were compared using the Chi-square test. Correlations between the SAF levels and lower-limb strength (STS), gait speed and balance (SLS and TUG) were evaluated using Spearman’s correlation analysis. Additionally, binomial logistic regression analysis was performed to assess independent associations between the SAF levels and lower-limb physical performance. Furthermore, a receiver-operating characteristic (ROC) curve analysis was performed to determine the optimal SAF cutoff value for predicting the risk of mobility decline. All statistical significance tests were two-tailed, and *p*-values < 0.05 were considered statistically significant.

## 3. Results

A total of 552 participants were included in the study, with a mean age of 76.33 ± 7.98 years. The mean SAF value was 2.92 ± 0.62 AU (Table 1).

Quartile group analysis based on the SAF values demonstrated significant differences in the age (*p* < 0.001), diabetes prevalence (*p* = 0.001), smoking status (*p* = 0.001), and ASMI (Appendicular Skeletal Muscle index; *p* = 0.026). Additionally, the comparison of the physical function across the quartile groups revealed that the group with the highest SAF values, Q4, exhibited a general decline in the physical performance when compared with the other groups (Table 2).

**Figure 2:**
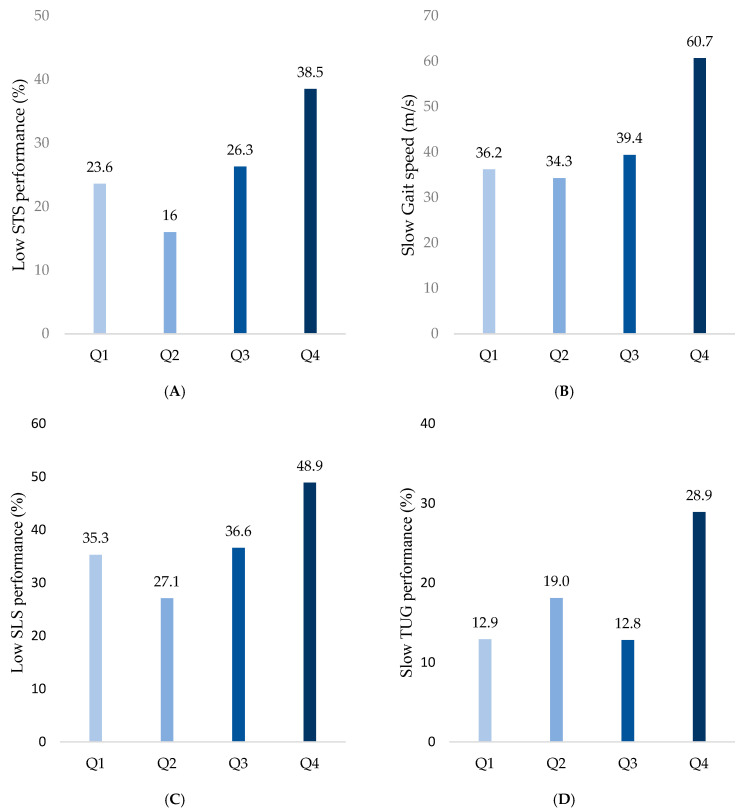
Comparison of physical performance decline among SAF quartiles. (**A**) Low STS performance was defined as the lowest 25th percentile in both sexes. (**B**) Slow gait speed was defined as ≤0.8 m/s. (**C**) Low SLS performance was defined as ≤5 s. (**D**) Slow TUG performance was defined as ≥13.5 s.

The frequency of low STS performance was highest in Q4 (38.5%), followed by Q1 (23.6%), Q2 (16%), and Q3 (26.3%) (*p* < 0.001; Figure 2A). The frequency of slow gait speed progressively increased with higher SAF values, recorded as 36.2%, 34.3%, 39.4%, and 60.7% in Q1, Q2, Q3, and Q4, respectively (*p* < 0.001; Figure 2B). Similarly, low SLS performance was observed more frequently in groups with higher SAF values, with proportions of 35.3%, 27.1%, 36.6%, and 48.9% in Q1, Q2, Q3, and Q4, respectively (*p* = 0.002; Figure 2C). Finally, slow TUG was observed in 12.9%, 19.0%,12.8%, and 28.9% of Q1, Q2, Q3, and Q4 groups, respectively, indicating decreases in the dynamic balance with increases in the SAF values (*p* = 0.001; Figure 2D).

Spearman’s correlation analysis revealed that the SAF-AGEs demonstrated significant negative correlations with STS (*r* = −0.211, *p* < 0.001), gait speed (*r* = −0.243, *p* < 0.001), and SLS (*r* = −0.201, *p* < 0.001). Additionally, TUG showed a significant positive correlation (*r* = 0.239, *p* < 0.001) (Table 3).

Logistic regression analysis confirmed that the SAF-AGEs level was an independent factor that was significantly associated with mobility decline. Compared with the Q1 group, the Q4 group had significantly higher odds of low STS performance (odds ratio (OR), 2.43; *p* = 0.006), slow gait speed (OR, 2.28; *p* = 0.002), low SLS performance (OR, 2.52; *p* = 0.001), and slow TUG (OR, 2.00; *p* = 0.035) (Table 4).

**Figure 3:**
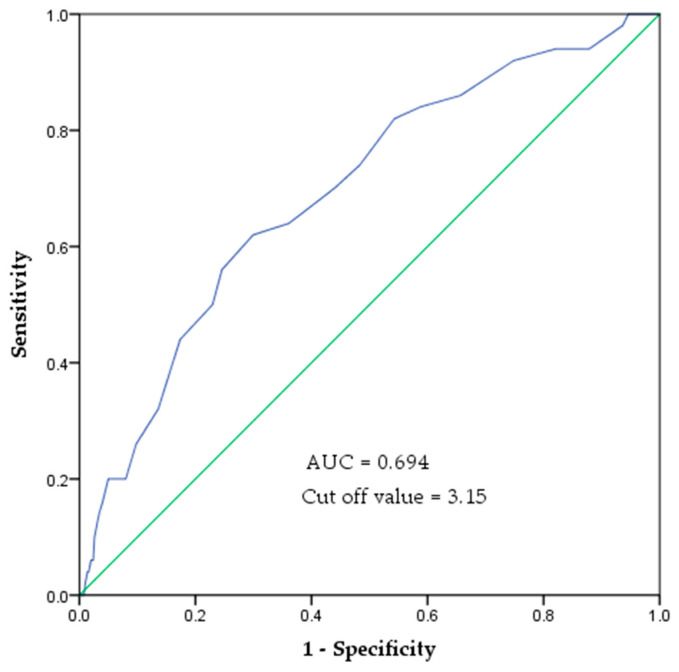
ROC curve of SAF values to indicate the risk of mobility decline. The area under the ROC curve was 0.694 (*p* < 0.001). The cut-off value is 3.15 AU, with a sensitivity of 0.62 and specificity of 0.70.

In the ROC curve analysis, the area under the curve (AUC) for predicting mobility decline based on SAF values was 0.694 (95% confidence interval (CI): 0.618–0.771). The optimal SAF cutoff value was 3.15, with a sensitivity of 0.62 and specificity of 0.70 (Figure 3).

## 4. Discussion

AGEs are a major cause of muscle strength decline, exerting negative effects on muscle tissues through various physiological pathways. AGEs form non-enzymatic cross-links with proteins, thereby altering the structure of extracellular matrix proteins comprising muscles and skeletal tissues. These structural changes negatively affect tissue stiffness and passive viscoelastic properties. Consequently, the altered physical properties of musculoskeletal tissues reduce force transmission from the muscle fibers, resulting in a decline in muscle strength and function [29]. Additionally, AGEs bind to the receptor for AGEs (RAGE), promoting inflammatory responses and oxidative stress, which contribute to muscle cell dysfunction and the aging of muscle cells [30]. However, AGEs accumulation is likely a consequence rather than the initial trigger of oxidative stress and inflammation. Minor but chronic oxidative stress and proinflammatory conditions create a favorable environment for the formation of AGEs, nitrosylated proteins, and lipids, which in turn propagate oxidative damage through radical chain reactions. This bidirectional relationship between oxidative stress and AGEs suggests a reinforcing cycle rather than a one-way causative mechanism. Considering these physiological mechanisms, the present study also observed an association between the SAF levels and a decline in lower-limb muscle strength. In particular, this study utilized the STS test to comprehensively assess the overall impact of SAF on lower-limb muscles. The STS test holds significant importance as it reflects not only the isometric strength of specific muscle groups, but also the overall lower limb strength, coordination, and proprioception [31]. Moreover, the STS test is a useful tool for evaluating not only muscle strength decline, but also neuromuscular integration and gait ability [32], thus demonstrating its value as a multifaceted measure for analyzing the broad effects of SAF on lower limb strength. This study contributes to a more comprehensive understanding of how AGE accumulation affects lower-limb muscle strength and functional mobility. Additionally, diabetes and smoking status were also significantly associated with higher SAF values. Previous studies have demonstrated that individuals with diabetes tend to exhibit greater AGEs accumulation due to prolonged hyperglycemia, which promotes glycation reactions. Similarly, smoking has been linked to increased oxidative stress, which accelerates AGEs formation. However, in this study, while these factors were significantly associated with SAF levels, they did not show a direct correlation with mobility decline. This suggests that AGEs accumulation may have independent effects on physical function beyond traditional metabolic risk factors.

Gait speed was also negatively affected by AGEs. Higher SAF values were significantly associated with reduced gait speed, and the gait speed of the upper quartile (Q4) group was noticeably lower than that of the other groups. AGEs may serve as key factors that contribute to impairments in gait mechanisms. Gait is a complex motor process that requires muscle coordination and adaptive neuromotor patterns. Muscle activation plays a crucial role during both the stance and swing phases of walking [33]. During the stance phase, the hip and knee extensors enhance the trunk stability in the initial posture, whereas the plantar flexors generate propulsion in the late stance phase to achieve forward acceleration. Additionally, to improve gait speed, the iliopsoas muscle is activated during the swing phase to accelerate the leg, followed by the deceleration of the hamstrings to ensure stable landing [34]. However, AGE accumulation may lead to muscle weakening and impaired neuromuscular control, resulting in a decreased gait speed [35,36]. Waqas et al. also found that an increase in the AGE levels was associated with a decline in gait speed in older adults, which is consistent with the findings of this study [37]. In contrast, Tabara et al. did not observe a significant correlation between the AGEs levels and gait speed [38]. This discrepancy may be attributed to the fact that Tabara et al. included a relatively younger sample with a mean age of 58 years, in which the cumulative effects of AGEs might not have been as pronounced as those in older populations. These findings suggest that the effects of the AGE levels on neuromuscular control and gait performance increase with age.

Balance can be defined as the ability to maintain the body’s center of gravity (CoG) within the base of support (BoS), and is generally categorized as static and dynamic balance [39]. Static balance refers to the ability to keep the CoG stable within the BoS while sitting or standing [40], whereas dynamic balance refers to the ability to maintain balance even when the CoG and BoS are moving [41]. Balance control requires the integration of the visual, vestibular, and proprioceptive sensory systems, along with sufficient muscle strength and coordination [42,43]. AGEs have been shown to negatively affect the neuromuscular control and cause muscle weakness. AGEs impair the efficiency of neuromuscular control by delaying pre-movement time and increasing the inefficient coactivation of antagonist muscles [44]. Therefore, the accumulation of AGEs contributes to both muscle weakness and impaired neuromuscular control. This negative impact of AGEs was also observed in the present study, as they were found to impair both static and dynamic balance. Although limited research has directly investigated the effects of AGE levels on balance, a recent study by Iida et al. found SAF values to be independently associated with an increased risk of falls [45]. Balance impairment, along with muscle weakness, is a key factor contributing to fall risk. Iida’s findings suggest that AGEs may impair balance ability, supporting the results of the present study. Therefore, AGEs can be considered a potential factor contributing to balance impairment.

The logistic regression analysis confirmed that SAF levels remained significantly associated with mobility decline even after adjusting for age, sex, BMI, diabetes, and smoking status. This indicates that AGEs accumulation in the skin is an independent risk factor for functional decline in older adults. According to previous studies, the key biomarkers that are significantly correlated with mobility decline in older adults include C-reactive protein (CRP), interleukin-6 (IL-6), and insulin-like growth factor (IGF-1) [46]. However, these biomarkers require blood sampling, which is invasive and impractical for routine examination. In contrast, SAF measurement offers a noninvasive and simple method, making it a practical and accessible tool for the early prediction of mobility decline in older adults. Notably, this study suggests that an SAF cutoff value of ≥3.15 AU may indicate an increased risk of mobility decline in older adults. In this study, the AUC was found to be 0.694 (95% CI: 0.618–0.771). However, because the AUC value was 0.694, which does not indicate a very high predictive power, there are limitations to using SAF values alone as a definitive indicator of mobility decline. Therefore, SAF measurements should be used in combination with physical function tests to better identify individuals at risk of mobility decline, and provide personalized management. In particular, SAF testing, owing to its simplicity, can be effectively utilized as a screening tool in community-based elderly care facilities, such as senior welfare and public health centers. Incorporating SAF measurements into routine assessments may help prevent mobility decline and contribute positively to health management in older adults.

This study had several limitations. First, it was a cross-sectional study; thus, the ability to establish a clear causal relationship between the AGE levels and factors related to mobility decline was limited. Therefore, future studies should adopt a longitudinal design to investigate the long-term effects of AGE accumulation on the decline of lower limb function. Second, the study participants were older adults residing in South Korea, which may limit the generalizability of the findings. Therefore, further studies with larger and more diverse samples including individuals from various ethnic groups are warranted to validate these results. Third, the SAF measurement method may be influenced by individual skin characteristics such as pigmentation and thickness, which could affect the accuracy of the results. To complement the SAF measurements, future studies should also measure and compare the serum AGE levels, such as carboxymethyllysine (CML), levels to improve the accuracy and reliability of the findings. Finally, Since AGEs formation is influenced by oxidative stress, environmental factors such as UV exposure and air pollutants, as well as dietary factors like carotenoid intake, may affect their accumulation. AS these variables were not controlled in this study, future research should consider these influences for a more comprehensive analysis.

## 5. Conclusions

This study comprehensively analyzed the impact of AGE accumulation in skin tissue on lower limb strength, gait speed, and balance in older adults. The findings demonstrate that higher SAF values are associated with decreased lower limb strength and gait speed, as well as significantly reduced balance abilities, indicating a potential negative association between SAF and mobility decline in older adults. Therefore, the SAF values may serve as a comprehensive screening tool for identifying the risk of mobility decline in older adults.

## Figures and Tables

**Table 1:** Clinical characteristics of study participants.

Total Population	
N	552
SAF, AU	2.92 ± 0.62
Age (years)	76.33 ± 7.98
Sex (men, %)	23
BMI (kg/m^2^)	24.49 ± 3.65
Diabetes status (Yes, %)	27
Smoke status (Yes, %)	12.6
ASMI (kg/m^2^)	6.54 ± 1.42
CC (cm)	33.78 ± 3.17

Note: SAF, AU = skin autofluorescence of advanced glycation end products, arbitrary units; BMI = body mass index; ASMI = Appendicular Skeletal Muscle index; CC = calf circumference.

**Table 2:** Differences in clinical parameters among quartiles of SAF.

	Q1	Q2	Q3	Q4	*p*
N	140	144	133	135	
SAF (AU)	2.25 ± 0.31	2.65 ± 0.18	3.07 ± 0.13	3.75 ± 0.39	<0.001 ***
Age (years)	76.39 ± 8.61	74.26 ± 8.01	75.86 ± 7.24	78.96 ± 7.27	<0.001 ***
Sex (men,%)	12.9	20.8	19.7	42.5	<0.001 ***
BMI (kg/m^2^)	24.96 ± 3.97	24.50 ± 3.64	24.15 ± 3.29	24.34 ± 3.65	0.333
Diabetes (Yes, %)	17.5	25.4	33.1	38.6	0.001 ***
Smoke (Yes, %)	8.3	11.3	10.2	20.7	0.001 ***
ASMI (kg/m^2^)	6.34 ± 1.32	6.58 ± 1.35	6.40 ± 1.43	6.83 ± 1.53	0.042 *
CC (cm)	33.87 ± 3.42	34.04 ± 3.16	33.45 ± 3.02	33.71 ± 3.04	0.517
STS (reps)	14.51 ± 4.88	15.25 ± 4.69	13.63 ± 5.04	11.96 ± 4.61	<0.001 ***
Gait speed (m/s)	0.87 ± 0.24	0.87 ± 0.26	0.85 ± 0.23	0.73 ± 0.25	<0.001 ***
SLS (s)	19.64 ± 19.26	21.66 ± 20.15	18.99 ± 20.67	12.88 ± 15.87	<0.001 ***
TUG (s)	9.64 ± 4.12	9.85 ± 5.27	10.35 ± 5.91	13.13 ± 8.29	<0.001 **

Note: SAF, AU = skin autofluorescence of advanced glycation end products, arbitrary units; BMI = body mass index; ASMI = Appendicular Skeletal Muscle index; CC = calf circumference; STS = sit to stand; SLS = single-leg stance; TUG = Timed Up and Go. Values are mean ± standard deviation or frequency. Statistical significance was assessed by Kruskal–Wallis or chi-square test. * *p* < 0.05, ** *p* < 0.01, *** *p* < 0.001.

**Table 3:** Correlation analysis between SAF levels and physical performance variables.

	SAF AGEs	Age	Sex (Men)	BMI	Diabetes	Smoke	ASMI	CC	STS	Gait Speed	SLS	TUG
SAF AGEs	1											
Age	0.178 ***	1										
Sex (men)	0.201 ***	0.085 *	1									
BMI	−0.114 **	0.088 *	−0.078	1								
Diabetes	0.181 ***	0.028	0.004	0.094 *	1							
Smoke	−0.119 **	−0.009	−0.529 ***	0.024	0.003	1						
ASMI	0.027	−0.177 ***	0.699 ***	0.511 ***	0.049	−0.393 ***	1					
CC	−0.110 **	−0.258 ***	0.294 ***	0.555 ***	0.003	−0.144 **	0.697 ***	1				
STS	−0.211 ***	−0.437 ***	−0.031	−0.051	−0.058	−0.006	0.084	0.196 ***	1			
Gait speed	−0.243 ***	−0.398 ***	−0.058	−0.095 *	−0.081	0.053	0.012	0.121 **	0.513 ***	1		
SLS	−0.201 ***	−0.559 ***	−0.025	−0.099 *	−0.117 **	−0.009	0.088 *	0.204 ***	0.508 ***	−0.523 **	1	
TUG	0.239 ***	0.517 ***	−0.021	0.111 **	0.107 *	0.041	−0.100 *	−0.204 ***	−0.625 ***	−0.787 ***	−0.608 ***	1

Note: BMI = body mass index; ASMI = Appendicular Skeletal Muscle index; STS = sit to stand; CC = calf circumference; SLS = single-leg stance; TUG = Timed Up and Go. * *p* < 0.05, ** *p* < 0.01, *** *p* < 0.001 using Spearman’s correlation analysis.

**Table 4:** Logistic regression analysis of physical performance outcomes by quartile groups.

	Low STS Performance		Slow Gait Speed		Low SLS Performance		Slow TUG	
	OR (95% CI)	*p*	OR (95% CI)	*p*	OR (95% CI)	*p*	OR (95% CI)	*p*
Q1	Reference		Reference		Reference		Reference	
Q2	1.10 (0.54–2.24)	0.794	1.17 (0.70–1.96)	0.563	1.33 (0.77–2.23)	0.311	1.93 (0.99–3.80)	0.055
Q3	1.61 (0.82–3.17)	0.166	1.13 (0.67–1.92)	0.637	1.56 (0.90–2.70)	0.113	0.90 (0.42–1.91)	0.778
Q4	2.43 (1.29–4.59)	0.006 **	2.28 (1.35–3.84)	0.002 ***	2.52 (1.43–4.46)	0.001 ***	2.00 (1.05–3.85)	0.035 *

Note: STS = sit to stand; SLS = single-leg stance; TUG = Timed Up and Go. Odds ratio (95% confidence interval) and *p* values were calculated by a logistic regression analysis adjusted for age, sex, BMI, diabetes, smoke status. Low STS performance was defined as the lowest 25th percentile in both sexes. Slow gait speed was defined as ≤0.8 m/s. Low SLS performance was defined as ≤5 s. Slow TUG performance was defined as ≥13.5 s. * *p* < 0.05, ** *p* < 0.01, *** *p* < 0.001.

## Data Availability

The data presented are available on request from the corresponding author.
